# Negative mood state in Kermanshah population during COVID-19 quarantine linked to low physical activity levels: a cross-sectional online survey study

**DOI:** 10.1038/s41598-023-48009-4

**Published:** 2023-11-23

**Authors:** Mohammad Azizi, Alireza Aghababa, Rastegar Hoseini, Hadi Rohani, Maghsoud Nabilpour, Fardin Moradi

**Affiliations:** 1 Department of Exercise Physiology, Faculty of Sport Sciences, Razi University, Kermanshah, Iran; 2 Department of Sport Psychology, Sport Sciences Research Institute, No. 3, 5th Alley, Miremad Street, Motahari Street, Tehran, Iran; 3 Department of Exercise Physiology, Sport Sciences Research Institute, Tehran, Iran; 4 Department of Exercise Physiology, Faculty of Educational Sciences and Psychology, University of Mohaghegh Ardabili, Ardabil, Iran; 5 Nutritional Sciences Department, Faculty of Nutritional Sciences and Food Technology, Kermanshah University of Medical Sciences, Kermanshah, Iran

**Keywords:** Health care, Medical research

## Abstract

One of the most significant consequences of the coronavirus disease (COVID-19) pandemic is the anxiety and stress it causes among the general population, which can be reduced by engaging in regular physical activity. The aim of this study was to estimate the levels of physical activity and mood state during the COVID-19 quarantine among the Kermanshah population. In this cross-sectional study, a total of 2471 subjects (1256 males and 1215 females) were selected in the population of Kermanshah in the west of Iran, using the convenience sampling method. Physical activity levels were assessed using the physical activity (PA) questionnaire short form (IPAQ-SF), and mood state was measured by the abbreviated form of the Iranian version of the standard POMS questionnaire (Bill Morgan 1979). Participants completed the online questionnaire between March 28th and May 20th, 2020. Descriptive statistics (mean, standard deviation, and percentage), and deductive (Chi-square and Spearman's correlation) were used for data analysis. our study found that the majority of participants reported decreased PA levels during the quarantine period. Specifically, 71.7% reported a decrease in moderate-intensity physical activity, 80.5% reported a decrease in high-intensity PA, and 71.3% reported a decrease in the total volume of PA. The results showed that there was a significant difference between the number of sessions (P=0.001), intensity (P=0.001), and duration of exercise (P=0.001) before and during the coronavirus. There was a significant positive relationship between low (r=0.93; P=0.001) and high (r=0.673; P=0.034) levels of PA and negative mood state. Additionally, there was a positive relationship between moderate PA level (r=0.82; P=0.001) and a positive mood states. The study suggests that the current quarantine has negatively affected the mood states of the participants. Overall, the study recommends regular PA to prevent COVID-19 while adhering to health and safety protocols.

## Introduction

The COVID-19 pandemic has brought about significant changes in daily life, including increased periods of quarantine and isolation^1,2^. As a result of the pandemic, there has been a growing concern regarding mental health and well-being across populations worldwide.^3^ Physical Activity (PA) has been considered as a means of reducing stress and anxiety, combating depression, and improving overall mental health^4,5^. However, the impact of COVID-19 quarantine on PA levels and mood state is not fully understood, particularly among the Kermanshah population in Iran^6,7^.

Recent scientific literature has examined the PA Level during COVID-19 confinement, with inconsistent results regarding the association between PA patterns and confinement conditions^8^. Lesser et al. (2020) reported that among modestly active adults, 40.5% experienced a decrease in PA while 33% experienced an increase; additionally, 22.4% of active adults became less active while 40.3% increased their activity during lockdown^9^. One study reported no change in PA levels or alterations in general health (GH) and mood state (a person's natural disposition and characteristics that affect their behavior, emotions, and social interactions. It is an inherent aspect of individuals that remain stable over time and tends to influence their motives and actions) among Iranian adults participating in team sports during the COVID-19 pandemic^10^, while others found that COVID-19 outbreak negatively impacted mood state and reduced GH and quality of life^11,12^.

Kermanshah province is located in the west of Iran, and it was one of the provinces severely affected by the COVID-19 outbreak. The government implemented strict quarantine measures to slow the spread of the virus, which significantly impacted daily life for the people of Kermanshah. PA levels were likely also affected, as access to outdoor activities was restricted during the quarantine period^13,14^. The impact of quarantine measures on PA levels and mood state among the Kermanshah population is therefore an important area to investigate. Given the current situation, this study aimed to investigate PA levels and mood state among the Kermanshah population during COVID-19 quarantine.

Specifically, this study aims to determine the relationship between PA levels and mood state during quarantine and identify potential predictors of PA and mood state.

## Methods

### Study design

This study is an applied research that employed available sampling method to investigate men and women residing in Kermanshah, Iran during the period between March 28th and May 20th, 2020. This period was characterized by a high infection rate of COVID-19 in Iran with an average of 9370 new cases per week and 139511 total cases^21^. The statistical population of the cross-sectional-descriptive study involved all Iranian adults aged 20-50 years residing in Kermanshah city, with the sample size estimated to be 400000 using the convenience sampling approach. An online questionnaire was distributed to the available samples using social media applications such as Telegram and WhatsApp, of which 2769 individuals completed the questionnaire. After excluding incomplete answers, a total of 2471 participants (1256 males and 1215 females) were deemed eligible for further analysis. Before conducting this study, the researchers obtained written informed consent from all participants, which included a detailed explanation of the aim, methodology, potential benefits, and risks associated with participation in the study. Participants were given the opportunity to ask questions about the study and were informed of their right to withdraw from the study at any time without any consequences. The researchers also ensured that participants' data was kept confidential and anonymous by using unique identification codes instead of personal information. By obtaining informed consent from participants, the researchers could ensure that the study was conducted in an ethical and transparent manner, while also respecting the rights and autonomy of the participants. The Ethics Committees of the Sport Sciences Research Institute of Iran approved the study under Protocol Number IR.SSRC.REC.1399.070 in conformity with the Declaration of Helsinki principles.

### Procedure

The online survey was anonymous, and participant identities were not attributable. An announcement, which included the link to the online survey, was published on all websites and communication networks of Kermanshah province. The online survey was disseminated via social media platforms such as Telegram, WhatsApp, and it was shared with personal contacts of the research group members and university students. Before beginning the questionnaire, the online survey form included a brief description of the study, its purpose, and the declarations of anonymity and confidentiality.

### Inclusion criteria

- Individuals who experienced quarantine during the COVID-19 pandemic.
- Individuals who provided complete information about their physical activity levels and mood state during quarantine.
- Individuals who did not have a history of mental illness or psychiatric treatment.
- Individuals who did not have physical disabilities or medical conditions that limit their ability to engage in physical activity.
- Individuals who are at least 18 years old and above.

### Exclusion criteria

- Individuals who did not experience quarantine during the COVID-19 pandemic.
- Individuals who did not participate in the online survey.
- Individuals who did not provide complete information about their physical activity levels and mood state during quarantine.
- Individuals who have physical disabilities or medical conditions that limit their ability to engage in physical activity.

The participants were recruited during the COVID-19 quarantine in Iran, a period during which the government's measures limited access to PA practices in all gyms, sports centers, and swimming pools, and prohibited any outdoor PA in public parks and gardens. A cleaning process was adopted, which included the removal of ineligible cases and multiple submissions by the same respondent, identification and handling of meaningless data, represented by invalid responses due to respondents' reluctance to provide valid responses and the lack of internal consistency of responses. The data collection tools consisted of the Physical Activity Questionnaire (IPAQ) and the mood state was measured by the Standard POMS questionnaire (Bill Morgan, 1979). To manage this last point, a threshold/cutoff value was calculated according to the IPAQ scoring protocol, as reported in the "Guidelines for Data Processing and Analysis of the IPAQ—Short and Long Forms," under the constraint of consistency of responses (http://www.ipaq.ki.se).

### Questionnaire

Since the questionnaire was administered to participants only once, the levels of PA for both conditions (before and during COVID-19) were assessed simultaneously. The online self-reporting questionnaire consisted of 31 questions investigating the respondents' PA practice in terms of frequency and duration of sitting, walking, moderate-intensity physical activities, and vigorous-intensity physical activities. The questionnaire in Appendix A was divided into nine sections, including demographic data (questions 2 and 3), anthropometric data (questions 4 and 5), PA before COVID-19 quarantine (questions 6 and 7), information relating to employment and residence during COVID-19 quarantine (questions 8 to 13), information before and during COVID-19 quarantine relating to vigorous-intensity PA (questions 14 to 17), moderate-intensity PA (questions 18 to 21), walking activity (questions 22 to 25), sedentary behaviors (questions 26 and 27), and additional information regarding the practice of PA during COVID-19 quarantine (questions 28 to 31). The mood state was measured by the Standard POMS questionnaire (Bill Morgan, 1979), which was translated and modified in some of the questions. The validity and reliability of the test were determined using correlation and the Cronbach Alpha test, which obtained a score of 0.87. The POMS questionnaire includes six mental states: Tension, depression, vitality, anger, fatigue, and confusion^15^.

### Data analysis

All statistical analyses were performed using the SPSS statistical software (version 21; SPSS Inc., Chicago, IL, USA) at a significant level of P< 0.05. The Kolmogorov–Smirnov test was used to evaluate the normality of distribution. The descriptive statistic method, including mean, standard deviation, and percent, and deductive methods such as Chi-square and Spearman's correlation, were used to analyze the data.

### Ethical approval

The Ethics Committees of the Sport Sciences Research Institute of Iran approved the study under Protocol Number IR.SSRC.REC.1399.070 in conformity with the Declaration of Helsinki principles.

### Compliance with ethics requirements

All procedures were under the ethical standards of the responsible committee on human experimentation (institutional and national) and with the Helsinki Declaration of 1975, as revised in 2008 (5). Informed consent was obtained from all patients included in the study.

### Consent to participate

Informed consent was obtained from all individual participants included in the study.

## Results

The baseline characteristics of the studied participants are presented in Table 1. In this study, we aimed to investigate the factors that contribute to the development and progression of PA. As part of this trial, we collected baseline characteristics from 2471 participants, including age, sex, marital status, Geographical location of lifeو mental health status, getting infected with COVID-19, educational level, and Job Status. Our results showed that the average age of the male and female participants was 27.87±5.011 and 25.12±6.49 years respectively, and there was a roughly equal distribution of male and female participants.

**Table 1:** Baseline characteristics of studied participants.

Variable	Male (n=1256)	Female (n=1215)
Age	27.87±5.011	25.12±6.49
Married	823	595
Single	405	600
Divorced	28	20
Urban	597	544
Rural	544	524
Suburbs	115	147
Positive mental states	302	404
Negative mental states	954	811
Getting infected	110	83
No infection	381	269
Not knowing	765	863
Observance	289	730
No observance	967	485
Education rate		
High school	195	145
Diploma	177	196
Associate degree	346	289
BSc	416	417
MSc	105	156
PhD	17	12
Job status		
Manual worker	137	29
Employee	76	33
Student	178	260
Part-time	163	99
Self-employed	256	88
Unemployed	446	706

*BSc* bachelor of sciences; *MSc* masters of sciences; *PhD* doctor of philosophy.

Table 2 shows that a significant difference was observed between the number of sessions, intensity, and duration of exercise before and during the coronavirus outbreak. Based on these results, the number, intensity, and duration of training sessions during the outbreak showed a significant decrease

**Table 2:** Number, intensity, and duration of sessions before and during the coronavirus between subjects.

Number of practice sessions	Before the coronavirus	During the coronavirus	Chi-squared test	P-value
Never	134	274	2153.023	0.001*
1 day	219	214		
2 days	255	305		
3 days	522	354		
4 days	328	172		
5 days	138	147		
6 days	127	80		
7 days	329	312		
Sometimes	419	613		
Total	2471	2471		
The intensity of training sessions				
Low	248	699	539.906	0.001*
Moderate	1041	1352		
High	997	349		
Very high	185	71		
Total	2471	2471		
Duration of training sessions				
Less than 30 min	519	839	479.021	0.001*
More than 30 min	1952	1632		
Total	2471	2471		

*Significantly number, intensity, and duration of sessions before and during the coronavirus; The value is calculated using Chi-Squared test.

The results in Table 3 show a significant negative relationship between positive mood and low PA levels (r = − 0.73; P = 0.002) and a significant positive relationship between positive mood and moderate PA levels (r = 0.82; P = 0.001). Also, there was no significant relationship between positive mood and high PA levels (r = 0.067; P = 0.331). In addition to these results, there was a significant positive relationship between negative mood and low levels of PA (r = 0.93; P = 0.001) and high levels of PA (r = 0.673; P = 0.034), and a significant negative relationship between negative mood and moderate PA levels (r = − 0.87; P = 0.001).

**Table 3:** The relationship between the PA levels and mental states.

Mental states	PA levels		
	Low	Moderate	High
Positive mood	R = − 0.73	r = 0.82	r = 0.321
	P = 0.002^¥^	P = 0.001^¥^	P = 0.331
Negative mood	r = 0.93	r = − 0.87	r = 0.673
	P = 0.001^¥^	P = 0.001^¥^	P = 0.034^¥^

^¥^Significant relationship between mental states with PA levels.

*Significant relationship between PA levels and mental states.

The value is calculated using Spearman's correlation coefficient test.

## Discussion

The results of the present study showed that the intensity of exercise, duration, and the number of sessions per week significantly decreased during the COVID-19 outbreak compared to before. While these results were not unexpected, the fear of infection may have limited various activities, including social activities and exercise, and may have even led to the closure of clubs after the outbreak. Furthermore, these changes in lifestyle, combined with not having fun leisure time, led to an increase in stress and anxiety levels in society.

However, the closure of physical activities has been linked to many diseases, worsening obesity and motor poverty^16^. Health researchers therefore recommend modifying workout routines to strengthen the immune system^16, 17^. Due to restrictions and closures of sports clubs to prevent the spread of coronavirus, attending clubs has become limited^18, 19^. To prevent the consequences of a sedentary lifestyle, such as obesity, home exercise and increased physical activities such as walking may be beneficial^16, 20^.

Furthermore, the results of the present study indicate that moderate levels of exercise activity significantly reduce negative mood states caused by the fear of COVID-19 and increase positive mood states. Being sedentary resulted in a significant increase in negative mood states and a significant decrease in positive mood states. In this study, a high level of PA was not only significantly related to increased positive mood states but also to increased negative mood states.

In general, PA is one of the most important contributors to physical and mental health. Exercise can improve the body's ability to fight infection by strengthening the immune system^18^. However, according to some study results, intense and competitive exercise may not be suitable during these conditions and can reduce the body's immune response, leading to a higher risk of COVID-19 infection^21, 22^. Most studies have highlighted the importance of avoiding a sedentary lifestyle, which is very common, especially among young people who spend excessive amounts of time on social media and networks. In addition to improving immune function and capacity, PA can help reduce anxiety and stress, improve mood, and manage underlying diseases such as diabetes, hypertension, and cardiovascular disease. Healthy adults should engage in at least 30 minutes of moderate-intensity exercise per day. The findings of the study suggest that PA levels were significantly lower during quarantine, which in turn had a negative impact on mood state among the Kermanshah population. This low level of PA is not surprising given the limitations on movement and exercise during quarantine periods. Inactivity may lead to physical and mental health issues, which is supported by the finding that mood state was negatively impacted by low PA levels.

The negative impact of low PA levels on mood state was seen in the study, with a significant negative correlation between PA levels and mood state. Participants who engaged in PA had a better mood state than those who did not. This finding is supported by previous research, which has shown that exercise can lead to increased feelings of well-being and positive mood.

The findings of this study have important implications for public health policy during quarantine periods. It is crucial to promote PA during quarantine to mitigate the negative impact on mental and physical health. This can include providing resources and guidance for home workout routines, as well as encouraging outdoor PA while adhering to social distancing measures.

Previous studies have shown that physical inactivity has become a significant public health concern during the COVID-19 pandemic^23, 24^. Several studies have reported a decrease in PA levels in both healthy individuals and people with chronic diseases during quarantine measures. For instance, a systematic review and meta-analysis showed that people who were under lockdown during the pandemic had significantly decreased PA levels than before the pandemic^25^. Similarly, Hansen et al. (2022) found evidence that PA levels decreased substantially during COVID-19 quarantine among the general population^26^. Furthermore, research has also shown that low PA levels during COVID-19 quarantine measures are associated with negative mood states. Maugeri et al. (2020) demonstrated that inactivity levels during COVID-19 confinement were positively correlated with depression and anxiety symptom severity^27^. Similarly, Aghababa et al. (2021) found that a lower level of PA was associated with increased levels of stress, anxiety, and depression among Iranian individuals during quarantine^28^.

The COVID-19 pandemic and the subsequent quarantine measures have led to increased periods of inactivity for many individuals, which can have profound effects on hormone levels in the body. Hormones play a vital role in various physiological processes, including mood regulation, stress response, metabolism, and immune function. Reduced physical activity and changes in lifestyle during quarantine can disrupt hormone levels, potentially impacting mental and physical health. During periods of inactivity and quarantine, stress hormones like cortisol may be affected. Chronic stress and reduced physical activity can lead to dysregulation of the hypothalamic-pituitary-adrenal (HPA) axis, resulting in abnormal cortisol levels. High cortisol levels, associated with chronic stress, can contribute to mood disorders, impaired immune function, and metabolic disturbances. A study by Wilkialis et al. (2021) found increased stress and cortisol levels among individuals in quarantine, suggesting a potential link between inactivity, quarantine, and dysregulated stress hormone levels^29^. Also, inactivity and reduced physical activity during quarantine can also influence metabolic hormones such as insulin and leptin. Prolonged periods of inactivity and sedentary behavior can exacerbate insulin resistance, increasing the risk of metabolic disorders like type 2 diabetes. Sallis et al. (2020) conducted a study that revealed reduced physical activity during quarantine was associated with increased insulin resistance and higher levels of leptin, highlighting the negative effects of inactivity on metabolic hormone levels^30^. Moreover, Physical activity is known to stimulate the release of mood-regulating hormones like serotonin, dopamine, and endorphins, promoting well-being and reducing symptoms of depression and anxiety. Therefore, reduced physical activity during quarantine may result in imbalances in these mood-regulating hormones. Lesser et al. (2020) found a significant increase in depressive symptoms among individuals with lower levels of physical activity during quarantine, suggesting a potential link between reduced physical activity, altered mood-regulating hormone levels, and negative mental health outcomes^9^. However, the COVID-19 pandemic and the associated periods of inactivity and quarantine can have significant effects on hormone levels in the body. Disruptions in stress hormones, metabolic hormones, and mood-regulating hormones are observed during these periods, potentially impacting mental and physical health. It is crucial to recognize the potential impact of inactivity and quarantine on hormone levels and take steps to mitigate these effects through regular physical activity and maintaining a healthy lifestyle.

Overall, these studies provide further support for the current study's findings that low PA levels during COVID-19 quarantine measure correlate with negative mood states. It suggests that prioritizing PA during quarantine measures may have potential benefits for promoting mental health and well-being during this pandemic.

The strengths of a study that focuses on the relationship between exercise and mood states during the COVID-19 pandemic . Firstly, the study provides a comprehensive analysis by considering factors such as exercise intensity, duration, frequency, closure of clubs, and increased stress and anxiety levels. This comprehensive data allows for a thorough understanding of the relationship between exercise and mood during quarantine. Secondly, the study's findings align with previous research, which enhances its credibility. Additionally, the study offers clear recommendations for public health policy during quarantine, emphasizing the importance of promoting physical activity to mitigate the negative impact on mental and physical health. Furthermore, the study mentions a large sample size, which enhances the generalizability of the findings. Lastly, the study acknowledges the limitations of intense and competitive exercise during the pandemic, demonstrating a balanced perspective. Overall, these strengths contribute to the study's credibility, relevance, and potential impact on public health policies during quarantine.

## Limitation

There are several limitations to the study titled "PA Levels and Mood State during COVID-19 Quarantine among the Kermanshah Population: A Cross-Sectional Online Survey Study." These may include:

- *Self-reported data*: The study relies on self-reported PA levels and mood state, which may be subject to recall bias and may not accurately reflect participants' true behavior or emotional state.
- *Limited generalizability*: The study is limited to a specific population (the Kermanshah population) and may not be generalizable to other populations with different cultural or socio-economic backgrounds.
- *Online survey limitations*: The survey was conducted online, so it may have excluded individuals who did not have access to the internet or who were less comfortable filling out an online survey.
- *Cross-sectional design*: The study uses a cross-sectional design, which means that it captures data at a single point in time and does not follow participants over time.
- *Confounding variables*: The study did not assess for potentially confounding variables such as medication use or pre-existing medical conditions that may impact PA or mood state during quarantine.

However, it is important to acknowledge these limitations in order to accurately interpret the study's findings and understand the scope of the research.

## Conclusion

The COVID-19 quarantine has had a discernible effect on the mood states of individuals in the Kermanshah population. Notably, the level of physical activity (PA) has been found to play a significant role in influencing mood during this challenging period. Higher levels of PA have been associated with improved mood states, highlighting the importance of promoting physical activity as a means of enhancing mental health and overall well-being. Future research should focus on exploring the long-term effects of PA on mood states in different populations, providing valuable insights into the potential benefits of regular exercise during times of quarantine.

## Data Availability

The datasets generated and/or analyzed during the current study are available from the corresponding author upon reasonable request.
