# Author Correction: Negative mood state in Kermanshah population during COVID-19 quarantine linked to low physical activity levels: a cross-sectional online survey study

**DOI:** 10.1038/s41598-024-55670-w

**Published:** 2024-03-04

**Authors:** Mohammad Azizi, Alireza Aghababa, Rastegar Hoseini, Hadi Rohani, Maghsoud Nabilpour, Fardin Moradi

**Affiliations:** 1 Department of Exercise Physiology, Faculty of Sport Sciences, Razi University, Kermanshah, Iran; 2 Department of Sport Psychology, Sport Sciences Research Institute, No. 3, 5th Alley, Miremad Street, Motahari Street, Tehran, Iran; 3 Department of Exercise Physiology, Faculty of Sport Sciences, Razi University, Kermanshah, Iran; 4 Department of Exercise Physiology, Sport Sciences Research Institute, Tehran, Iran; 5 Department of Exercise Physiology, Faculty of Educational Sciences and Psychology, University of Mohaghegh Ardabili, Ardabil, Iran; 6 Nutritional Sciences Department, Faculty of Nutritional Sciences and Food Technology, Kermanshah University of Medical Sciences, Kermanshah, Iran

Correction to: *Scientific Reports* 10.1038/s41598-023-48009-4, published online 23 November 2023

The original version of this Article contained errors.

In the original version of this article, the name of Maghsoud Nabilpour was incorrectly given as Maghsoud Nabilpoor.

Furthermore, the original version of this Article contained an error in Affiliation 5.

Affiliation 5

“5M. A of Sport Physiology, Department of Sport Sciences, Imam Khomeini International University, Qazvin, Iran.”

now reads:

Affiliation 5

“Department of Exercise Physiology, Faculty of Educational Sciences and Psychology, University of Mohaghegh Ardabili, Ardabil, Iran.”

In addition, the corresponding author email address was incorrect. Correspondence and requests for materials should be addressed to alirezaaghababa@yahoo.com.

The original Article has been corrected.

